# Current status and trends in ERCP and post-ERCP pancreatitis in Japan: a nationwide observational study

**DOI:** 10.1007/s00535-025-02254-8

**Published:** 2025-05-02

**Authors:** Tomoo Manaka, Tetsuya Takikawa, Kunio Tarasawa, Kazuhiro Kikuta, Ryotaro Matsumoto, Yu Tanaka, Takanori Sano, Shin Hamada, Shin Miura, Kiyoshi Kume, Kenji Fujimori, Kiyohide Fushimi, Atsushi Masamune

**Affiliations:** 1 Division of Gastroenterology, Tohoku University Graduate School of Medicine, 1-1 Seiryo-machi, Aoba-ku, Sendai, 980-8574 Japan; 2 Department of Health Administration and Policy, Tohoku University Graduate School of Medicine, 2-1 Seiryo-machiachi, Aoba-ku, Sendai, 980-8575 Japan; 3 Department of Health Policy and Informatics, Institute of Science Tokyo, S1560/S1568 M&D Tower 1-5-45 Yushima, Bunkyo-ku, Tokyo, 113-8519 Japan

**Keywords:** Diagnosis procedure combination, Endoscopic retrograde cholangiopancreatography, Post-ERCP pancreatitis, Non-steroidal anti-inflammatory drugs, Protease inhibitors

## Abstract

**Background:**

Endoscopic retrograde cholangiopancreatography (ERCP) is indispensable for the management of biliary and pancreatic diseases but carries a high risk of post-ERCP pancreatitis (PEP). This study aimed to clarify the current status and temporal trends of ERCP and PEP in Japan, including preventive measures.

**Methods:**

We conducted a retrospective, population-based cohort study using the Diagnosis Procedure Combination database from April 1, 2016, to March 31, 2023. Trend analyses were performed for ERCP, PEP, nonsteroidal anti-inflammatory drugs (NSAIDs), and protease inhibitors. Additionally, factors associated with PEP and severe PEP were evaluated.

**Results:**

Among the 1,073,513 ERCP cases, PEP and severe PEP incidences were 85,212 (7.9%) and 4841 cases (0.5%), respectively. The mortality rate was 0.5% for severe PEP and 0.2% for non-severe cases. The number of ERCP procedures and the proportion of therapeutic ERCP increased over time. The incidence of PEP declined from 9.1% in the fiscal year 2016–2017 to 6.4% in the fiscal year 2022, while the incidence of severe PEP decreased from 0.5 to 0.33% over the same period. The usage rate of rectal NSAIDs increased from 16.4 to 27.6%, whereas that of protease inhibitors decreased from 70.5 to 53.5%. The administration of rectal NSAIDs at doses of 20–25 mg and 50 mg was associated with a reduced risk of severe PEP.

**Conclusions:**

The number of ERCP procedures and the proportion of therapeutic ERCP have increased, whereas the incidences of PEP and severe PEP have decreased. Rectal NSAIDs may prevent the progression of PEP to severe disease.

**Supplementary Information:**

The online version contains supplementary material available at 10.1007/s00535-025-02254-8.

## Introduction

Endoscopic retrograde cholangiopancreatography (ERCP) is indispensable for the diagnosis and treatment of biliary and pancreatic diseases. However, ERCP is technically challenging and carries a higher risk of adverse events than other common endoscopic procedures [1–3]. Among these, post-ERCP pancreatitis (PEP) is the most frequent and can be life-threatening. A recent systematic review and meta-analysis reported overall incidences of PEP, severe PEP, and mortality as 10.2%, 0.5%, and 0.2%, respectively [4]. Although various preventive measures have been explored, PEP cannot be entirely prevented and remains a major concern in ERCP procedures.

The landscape of ERCP and PEP has changed over recent decades. Advances in noninvasive imaging modalities, such as magnetic resonance cholangiopancreatography and endoscopic ultrasonography, have reduced the need for diagnostic ERCP. Consequently, the proportion of therapeutic ERCP has increased, potentially elevating the risk of PEP [5, 6]. The efficacy of preventive measures, such as prophylactic pancreatic stents, rectal non-steroidal anti-inflammatory drugs (NSAIDs), and aggressive hydration, has been demonstrated, contributing to a potential reduction in PEP incidence [2, 3, 7–9]. Several meta-analyses of randomized controlled trials have shown that protease inhibitors (PIs), which have been traditionally used in Japan, do not provide a significant preventive effect against PEP [10–12]. While studies have reported an increasing trend in PEP incidence from 2000 to the mid-2010s [6, 13], recent trends remain unclear. Furthermore, few studies have investigated trends in the use of preventive measures. The first clinical practice guidelines for PEP in Japan were published in 2015 [14], and their implementation is expected to have contributed to a decline in PEP incidence.

The Diagnosis Procedure Combination (DPC) is a comprehensive payment system for medical services, primarily implemented in acute-care hospitals. More than 1700 hospitals, including 82 university hospitals, have adopted the DPC system. The DPC database contains extensive information, including patient demographics, comorbidities at admission, disease-specific disability or severity scores, discharge status, diagnoses coded using the International Classification of Diseases, 10th revision (ICD-10), daily administered medications, and medical procedures performed during hospitalization [15, 16]. As one of the most widely utilized sources of real-world data in Japan, the DPC database plays a crucial role in understanding the healthcare landscape and addressing clinical issues [15, 16]. Using this database, the present study aimed to elucidate the current status and temporal trends of ERCP, PEP, and preventive measures in Japan.

## Methods

### Study design and data source

This retrospective, population-based cohort study was conducted using the DPC database. We analyzed data from the DPC database maintained by the DPC Research Group, which includes approximately 1100 facilities and covers over 90% of all tertiary-care emergency hospitals in Japan, reflecting real-world clinical practice in the country [17–20].

This study was conducted in accordance with the principles of the Declaration of Helsinki and was approved by the Ethics Committee of Tohoku University Graduate School of Medicine (approval number: 2023-1-944-1). The requirement for written informed consent was waived due to the anonymized nature of the data.

### Study population

We extracted all hospitalized cases that underwent ERCP between April 1, 2016, and March 31, 2023, from the DPC database. ERCP procedures were identified using the Medical Intervention Classification master code [21]. Diagnostic ERCP included 160,093,970 and 170,015,670 (cholangiography and pancreatography) and 160,161,170 and 170,020,370 (cholangioscopy and pancreatoscopy). Therapeutic ERCP included the procedures identified the procedure codes shown in Online Resource 1.

### Data collection

We collected data on sex, age, body mass index (BMI), Charlson Comorbidity Index (CCI) score, and discharge status. For BMI, values of 0 or Z-scores ≥ 3 or < -3 were excluded as outliers [22]. We also identified cases in which rectal NSAIDs, PIs, and antibiotics were administered on the day of the ERCP. All types of rectal NSAIDs recorded in the DPC database were extracted. PIs included gabexate mesilate, ulinastatin, and nafamostat mesilate. Antibiotics included carbapenems, first- to fourth-generation cephalosporins, and beta-lactam/beta-lactamase inhibitor combinations.

### Definitions

#### PEP

Among the cases who underwent ERCP, PEP was identified when recorded as a comorbidity at admission or a complication during hospitalization in Japanese text data. Other cases who underwent ERCP were classified as non-PEP cases.

#### Severe acute pancreatitis (AP)

For patients hospitalized with AP, the DPC database provides prognostic factors and computed tomography grades used to determine the severity of AP according to the Japanese severity criteria [20, 23]. However, since PEP is recorded as a comorbidity or complication, its severity is not routinely recorded. Therefore, based on previous reports [24, 25], severe PEP was defined as the occurrence of any of the following events within 30 days after ERCP: admission to the intensive care unit, use of a mechanical ventilator, use of dialysis, development of acute renal failure, multiple organ failure, respiratory failure, disseminated intravascular coagulation, or development of shock symptoms. These events were identified using the Medical Intervention Classification Master code, ICD-10 codes, and disease name text.

#### PEP-related mortality

PEP-related mortality was defined as death in patients with PEP whose primary diagnosis or the condition requiring the highest utilization of medical resources was either PEP or AP. In the DPC database, mortality outcomes are categorized as either “mortality due to the condition requiring the highest utilization of medical resources” or “mortality due to other causes.” However, the latter category does not provide detailed information on the specific cause of death.

### Statistical analysis

Continuous variables are presented as means with standard deviations (SDs), while categorical variables are expressed as numbers and percentages. For trend analysis, the Cochran–Armitage test was used for categorical variables, and the Jonckheere–Terpstra test was used for continuous variables. Trend analyses were conducted in 2-year groups from fiscal year (FY) 2016 to FY 2021, with FY 2022 analyzed as a single-year group. Comparisons between two groups were performed using the Student’s *t*-test for continuous variables and the chi-square test or Fisher’s exact test for categorical variables.

Univariate and multivariate logistic regression models were used to evaluate factors associated with PEP and severe PEP incidence. In the multivariate analysis, variables with a *P*-value < 0.2 in the univariate analysis were included. Multicollinearity among the variables was assessed by calculating variance inflation factors. Additionally, propensity score (PS) matching was performed to adjust for patient-related variables when evaluating factors associated with PEP and severe PEP. The adjustment included the following variables: age, sex, BMI, and CCI score. Matching was conducted in a 1:1 ratio with a caliper width of 0.2. The appropriateness of matching was evaluated using the C-statistic. The balance of covariates was assessed using standardized differences, and significant imbalance was defined as an absolute standardized difference ≥ 0.1 [17].

All statistical analyses were performed using JMP Pro 17 (SAS Institute Inc., Cary, NC, USA) and R version 4.4.0 for Windows (R Foundation for Statistical Computing, Vienna, Austria). Statistical significance was set at a two-sided *P*-value of < 0.05.

## Results

### Characteristics of enrolled cases

**Fig. 1:**
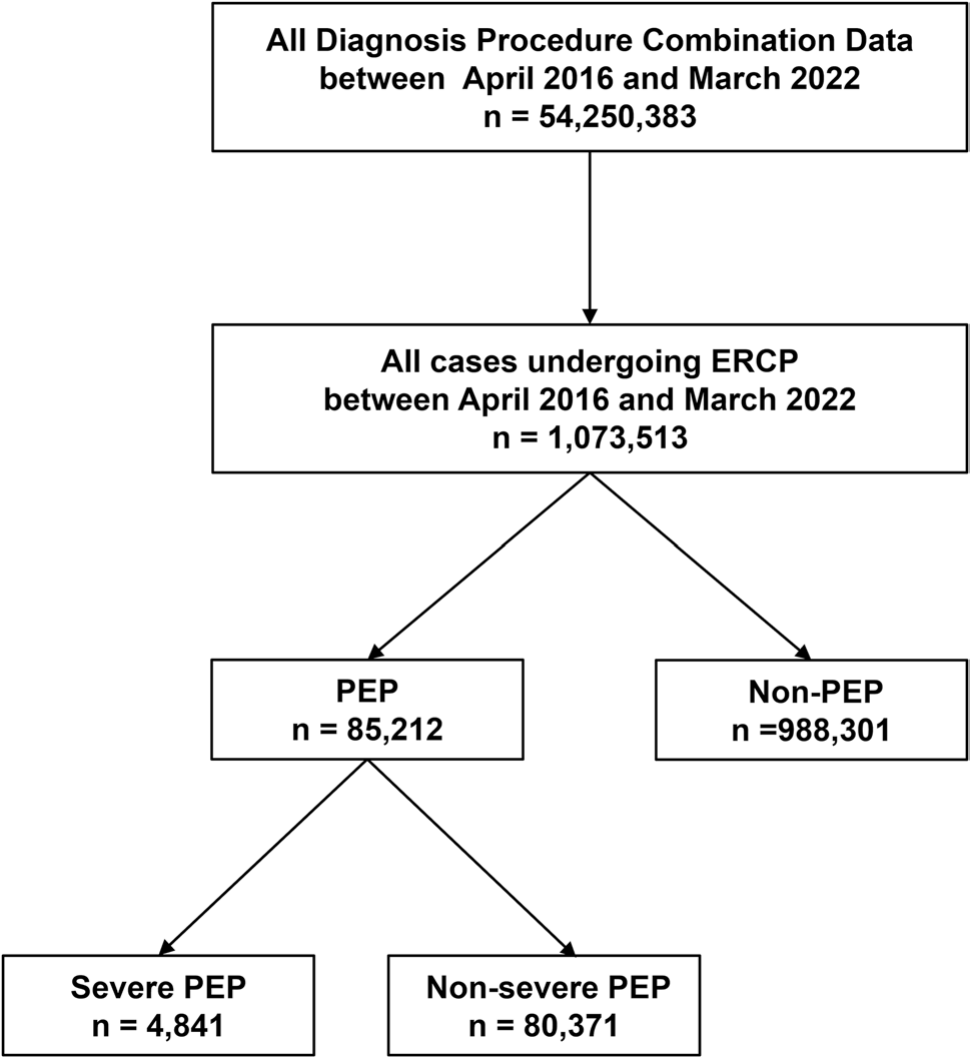
Flowchart of the selection process. *ERCP* Endoscopic retrograde cholangiopancreatography, *PEP* post-ERCP pancreatitis

Figure 1 shows the flowchart of the selection process used in this study. A total of 54,250,383 cases were recorded in the DPC database between April 1, 2016, and March 31, 2023. Among them, 1,073,513 cases who underwent ERCP were analyzed (Table 1). The mean age (SD) was 74.0 (13.2) years, and 624,261 (58.2%) cases were male. Diagnostic ERCP was performed in 107,441 (10.0%) cases, whereas therapeutic ERCP was much more common (966,072 cases; 90.0%). Rectal NSAIDs, PIs, and antibiotics were administered in 233,864 (21.8%), 680,197 (63.4%), and 722,470 (67.3%) cases, respectively. PEP and severe PEP incidences were 85,212 (7.9%) and 4841 (0.5%) cases, respectively. Among the 85,212 (4841 severe) cases of PEP, 77,888 cases (4397 severe) were associated with therapeutic ERCP, whereas 7324 cases (444 severe) were associated with diagnostic ERCP. The primary diagnosis was PEP in 1283 (30 severe) cases and AP in 357 (36 severe). The highest utilization of medical resources was attributed to PEP in 1220 (26 severe) cases and to AP in 316 (31 severe).

**Table 1:** Characteristics of all cases undergoing ERCP

Age, mean (SD), years	74.0 (13.2)
Sex, Male, n (%)	624,261 (58.2)
BMI (kg/m^2^)^a^	
< 18.5, n (%)	144,260 (14.1)
≥ 18.5, < 25, n (%)	654,776 (64.2)
≥ 25, n (%)	221,019 (21.7)
CCI	
0, n (%)	484,022 (45.1)
1–2, n (%)	437,131 (40.7)
≥ 3, n (%)	152,360 (14.2)
Type of ERCP	
Diagnostic ERCP, n (%)	107,441 (10.0)
Therapeutic ERCP, n (%)	966,072 (90.0)
Use of rectal NSAIDs, n (%)	233,864 (21.8)
12.5 mg, n (%)	4429 (0.4)
20–25 mg, n (%)	115,573 (10.8)
50 mg, n (%)	113,862 (10.6)
Use of protease inhibitors, n (%)	680,197 (63.4)
Gabexate mesilate, n (%)^b^	184,645 (17.2)
Ulinastatin, n (%)^b^	245,554 (22.9)
Nafamostat mesilate, n (%)^b^	290,492 (27.1)
Use of antibiotics, n (%)	722,470 (67.3)
PEP, n (%)	85,212 (7.9)
Severe PEP, n (%)	4841 (0.5)
PEP-related mortality, n (%)	202 (0.02)

*BMI*, body mass index; *CCI*, Charlson Comorbidity Index; *ERCP*, endoscopic retrograde cholangiopancreatography; *NSAIDs*, non-steroidal anti-inflammatory drugs; *PEP*, post-ERCP pancreatitis; *SD*, standard deviation

^a^Data from 1,020,055 cases

^b^Duplicate cases are included

During hospitalization, 2254 (2.6%) patients with PEP died. However, PEP-related mortality was observed in only 202 (0.2%) cases of PEP. The mortality rate was 0.5% (29/4841) for severe PEP and 0.2% (173/80,371) for non-severe PEP. The mortality rate was higher in severe PEP than in non-severe cases (*P* < 0.001). Among the 202 fatal cases, the primary diagnosis was PEP in 114 and AP in 19 (Online Resource 2). In the remaining 69 fatal cases, either PEP (66 cases) or AP (3 cases) was the condition that required the highest utilization of medical resources. Mortality was attributed to the condition that required the highest utilization of medical resources in 104 cases (PEP in 85 cases, AP in 14, DIC in 2, sepsis in 2, and pancreatic pseudocyst in 1). In the remaining 98 fatal cases, mortality was attributed to a condition other than that which required the highest utilization of medical resources, and the exact cause of mortality was not recorded. Among them, primary diagnosis was PEP in 54 cases, AP in 7, and others in 37.

### Temporal trends in ERCP

**Fig. 2:**
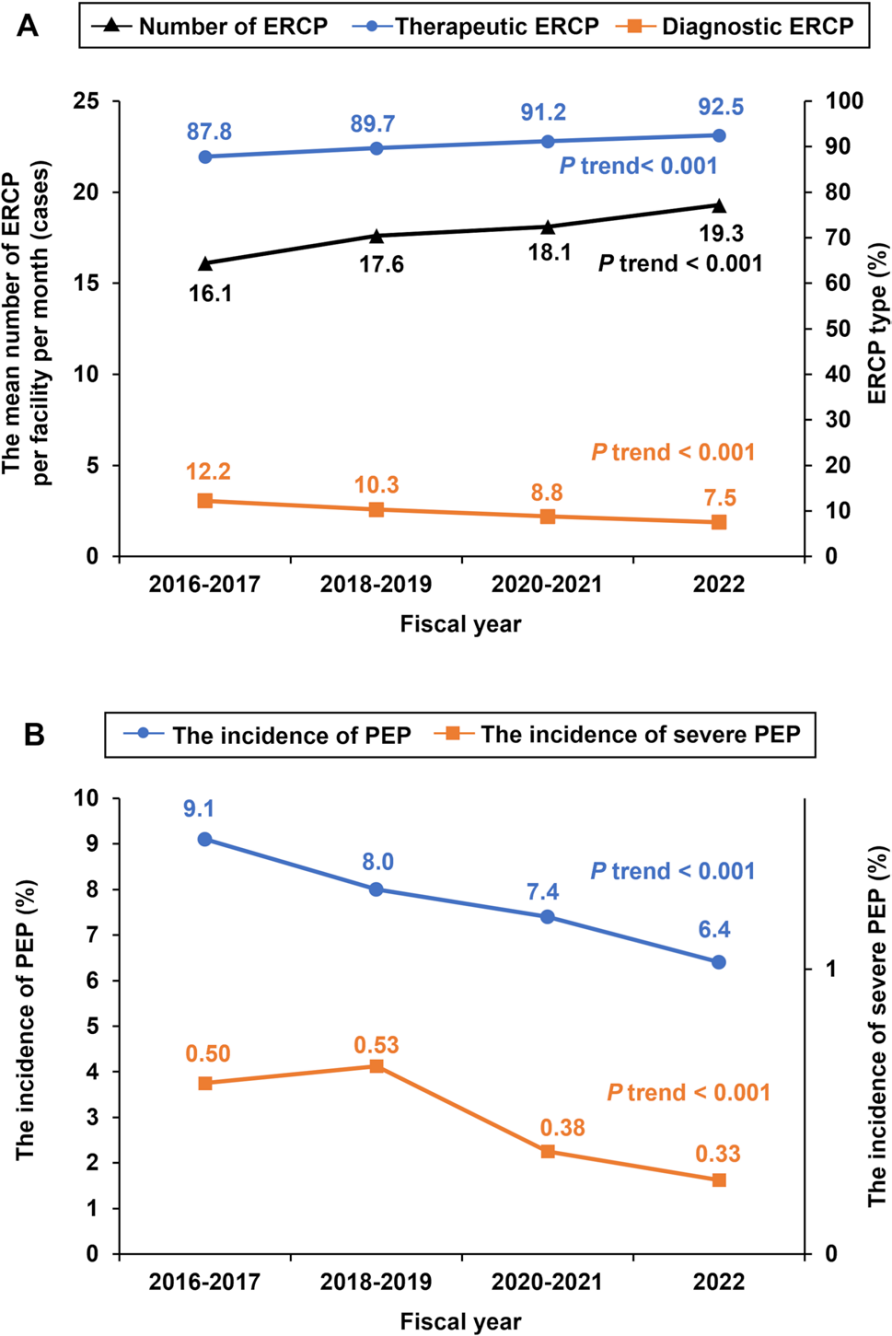
Trends in ERCP procedures and PEP incidence. **A** The number of ERCP procedures and the proportion of therapeutic ERCP showed increasing trends (*P* for trend < 0.001). **B** The incidence of PEP and severe PEP showed decreasing trends (*P* for trend < 0.001). *ERCP* Endoscopic retrograde cholangiopancreatography, *PEP* post-ERCP pancreatitis

The average number of ERCP procedures per facility per month increased from 16.1 in FY 2016–2017 to 19.3 in FY 2022. The proportion of therapeutic ERCP procedures also increased from 87.8% in FY 2016–2017 to 92.5% in FY 2022 (Fig. 2A and Table 2). A detailed overview of temporal trends in ERCP is provided in Table 2. The average age of patients undergoing ERCP gradually increased over time in both men and women. The usage rate of rectal NSAIDs, primarily at doses of 20–25 mg and 50 mg, increased from 16.4% in FY 2016–2017 to 27.6% in FY 2022. In contrast, the usage rate of PIs declined from 70.5% in FY 2016–2017 to 53.5% in FY 2022.

**Table 2:** Trends in ERCP among all cases from 2016 to 2022

Fiscal year	2016–2017	2018–2019	2020–2021	2022	*P* value for trend
The total number of ERCP, n	300,695	305,750	314,959	152,109	–
The number of ERCP per facility per month, mean (SD)	16.1 ± 15.6	17.6 ± 12.8	18.1 ± 16.3	19.3 ± 17.6	< 0.001
Age, mean (SD), years					
Total	73.4 (13.2)	73.9 (13.2)	74.4 (13.1)	74.6 (13.1)	< 0.001
Male	72.0 (12.5)	72.6 (12.5)	73.0 (12.5)	73.2 (12.4)	< 0.001
Female	75.2 (13.9)	75.8 (13.9)	76.3 (13.8)	76.4 (13.7)	< 0.001
Sex, Male, n (%)	174,067 (57.9)	177,960 (58.2)	183,420 (58.2)	88,814 (58.4)	< 0.001
BMI (kg/m^2^)^a^					
< 18.5, n (%)	40,799 (14.3)	41,219 (14.2)	41,838 (14.0)	20,404 (14.1)	0.005
≥ 18.5, < 25, n (%)	184,548 (64.6)	186,322 (64.1)	191,385 (63.9)	92,521 (64.0)	< 0.001
≥ 25, n (%)	60,193 (21.1)	62,943 (21.7)	66,174 (22.1)	31,709 (21.9)	< 0.001
CCI					
0, n (%)	134,618 (44.8)	137,032 (44.8)	141,607 (45.0)	70,765 (46.5)	< 0.001
1–2, n (%)	122,235 (40.6)	124,751 (40.8)	128,755 (40.9)	61,390 (40.4)	0.46
≥ 3, n (%)	43,842 (14.6)	43,967 (14.4)	44,597 (14.1)	19,954 (13.1)	< 0.001
ERCP type					
Diagnostic ERCP, n (%)	36,718 (12.2)	31,578 (10.3)	27,769 (8.8)	11,376 (7.5)	< 0.001
Therapeutic ERCP, n (%)	263,977 (87.8)	274,172 (89.7)	287,190 (91.2)	140,733 (92.5)	< 0.001
Use of rectal NSAIDs, n (%)	49,180 (16.4)	64,482 (21.1)	78,232 (24.8)	41,970 (27.6)	< 0.001
12.5 mg, n (%)	1275 (0.42)	1326 (0.43)	1299 (0.41)	529 (0.35)	< 0.001
20–25 mg, n (%)	24,246 (8.1)	31,807 (10.4)	38,532 (12.2)	20,988 (13.8)	< 0.001
50 mg, n (%)	23,659 (7.9)	31,349 (10.3)	38,401 (12.2)	20,453 (13.4)	< 0.001
Use of protease inhibitors, n (%)	211,914 (70.5)	197,464 (64.6)	189,394 (60.1)	81,425 (53.5)	< 0.001
Gabexate mesilate, n (%)^b^	64,643 (21.5)	52,685 (17.2)	47,092 (15.0)	20,225 (13.3)	< 0.001
Ulinastatin, n (%)^b^	75,609 (25.1)	70,601 (23.1)	70,144 (22.3)	29,200 (19.2)	< 0.001
Nafamostat mesilate, n (%)^b^	86,500 (28.8)	86,040 (28.1)	82,333 (26.1)	35,619 (23.4)	< 0.001
Use of antibiotics, n (%)	191,029 (63.5)	205,243 (67.1)	218,868 (69.5)	107,330 (70.6)	< 0.001
PEP, n (%)	27,493 (9.1)	24,569 (8.0)	23,340 (7.4)	9810 (6.4)	< 0.001
Severe PEP, n (%)	1508 (0.50)	1619 (0.53)	1212 (0.38)	502 (0.33)	< 0.001
PEP-related mortality, n (%)	44 (0.01)	73 (0.02)	60 (0.02)	25 (0.02)	0.68

*BMI*, body mass index; *CCI*, Charlson Comorbidity Index; *ERCP*, endoscopic retrograde cholangiopancreatography; *NSAIDs*, non-steroidal anti-inflammatory drugs; *PEP*, post-ERCP pancreatitis; *SD*, standard deviation

^a^Data from 1,020,055 cases

^b^Duplicate cases are included

### Temporal trends in PEP

The characteristics of PEP cases are summarized in Online Resource 3. Both PEP incidence and severe PEP incidence showed a decreasing trend (Fig. 2B and Table 2). The incidence of PEP declined from 9.1% in FY 2016–2017 to 6.4% in FY 2022, whereas the incidence of severe PEP decreased from 0.50 to 0.33% over the same period. The trend in ERCP among PEP cases was similar to that observed in all ERCP cases (Online Resource 4). Patient age, the proportion of therapeutic ERCP procedures, and the usage rate of rectal NSAIDs at doses of 20–25 mg and 50 mg showed upward trends. In contrast, the usage rate of PIs and the incidence of severe PEP demonstrated decreasing trends.

### Comparison between PEP and non-PEP cases

We compared the characteristics of PEP and non-PEP cases (Table 3). Compared with non-PEP cases, patients with PEP were older (74.3 vs. 74.0 years), had a lower proportion of males (54.0% vs. 58.5%), and had a higher proportion of therapeutic ERCP procedures (91.4% vs. 89.9%). The usage rate of rectal NSAIDs was higher in PEP cases than in non-PEP cases (23.5% vs. 21.6%), although no significant difference was observed for the 50 mg dose. Similarly, the usage rate of PIs was higher in PEP cases than that in non-PEP cases (81.1% vs. 61.8%).

**Table 3:** Comparison of characteristics between PEP and non-PEP

	Non-PEP cases (n = 988,301)	PEP cases (n = 85,212)	*P* value
Age, mean (SD), years	74.0 (13.2)	74.3 (13.2)	< 0.001
Sex, Male, n (%)	578,263 (58.5)	45,998 (54.0)	< 0.001
BMI (kg/m^2^)^a^			
< 18.5, n (%)	134,043 (14.3)	10,217 (12.6)	0.03
≥ 18.5, < 25, n (%)	602,920 (64.2)	51,856 (64.1)	< 0.001
≥ 25, n (%)	202,156 (21.5)	18,863 (23.3)	0.02
CCI			
0, n (%)	444,272 (44.9)	39,750 (46.7)	< 0.001
1–2, n (%)	402,852 (40.8)	34,279 (40.2)	0.002
≥ 3, n (%)	141,177 (14.3)	11,183 (13.1)	< 0.001
ERCP type			
Diagnostic ERCP, n (%)	100,117 (10.1)	7324 (8.6)	< 0.001
Therapeutic ERCP, n (%)	888,184 (89.9)	77,888 (91.4)	< 0.001
Use of rectal NSAIDs, n (%)	213,869 (21.6)	19,995 (23.5)	< 0.001
12.5 mg, n (%)	3900 (0.4)	529 (0.6)	< 0.001
20–25 mg, n (%)	105,132 (10.6)	10,441 (12.3)	< 0.001
50 mg, n (%)	104,837 (10.6)	9025 (10.6)	0.88
Use of protease inhibitors, n (%)	611,080 (61.8)	69,117 (81.1)	< 0.001
Gabexate mesilate, n (%)^b^	167,208 (16.9)	17,437 (20.5)	< 0.001
Ulinastatin, n (%)^b^	217,836 (22.0)	27,718 (32.5)	< 0.001
Nafamostat mesilate, n (%)^b^	261,284 (26.4)	29,208 (34.3)	< 0.001
Use of antibiotics, n (%)	665,724 (67.4)	56,746 (66.6)	< 0.001

*BMI*, body mass index; *CCI*, Charlson Comorbidity Index; *ERCP*, endoscopic retrograde cholangiopancreatography; *NSAIDs*, non-steroidal anti-inflammatory drugs; *PEP*, post-ERCP pancreatitis; *SD*, standard deviation

^a^Data from 939,119 non-PEP and 80,936 PEP cases

^b^Duplicate cases are included

We then analyzed the factors associated with PEP. Variance inflation factors ranged from 1.01 to 1.04, indicating no multicollinearity. In the multivariate analysis, age ≥ 65 years (odds ratio [OR] = 1.07), BMI ≥ 25 kg/m^2^ (OR = 1.07), therapeutic ERCP (OR = 1.17), use of rectal NSAIDs at doses of 12.5 mg (OR = 1.59), 20–25 mg (OR = 1.26), and 50 mg (OR = 1.17), and use of PIs (OR = 2.70) were associated with an increased risk of PEP, whereas male sex (OR = 0.83), BMI < 18.5 kg/m^2^ (OR = 0.88), CCI score of 1–2 (OR = 0.96) and ≥ 3 (OR = 0.92), and use of antibiotics (OR = 0.89) were associated with decreased risk of PEP (Online Resource 5). Given the differences in patient-related factors, we performed PS matching for age, sex, BMI, and CCI score, achieving a C-statistic of 0.5 and an absolute standardized difference of < 0.001 for each variable (Online Resource 6). After PS matching, therapeutic ERCP (OR = 3.63), use of rectal NSAIDs at doses of 12.5 mg (OR = 1.64), 20–25 mg (OR = 1.18), and 50 mg (OR = 1.27), use of PIs (OR = 2.30), and use of antibiotics (OR = 1.74) were identified as risk factors for PEP (Online Resource 7).

### Comparison between severe PEP and non-severe PEP cases

Table 4 compares severe and non-severe PEP cases. Compared with non-severe PEP cases, patients with severe PEP were associated with older age (75.0 vs. 74.3 years), male sex (58.8% vs. 53.7%), and a higher CCI score (≥ 3: 25.5% vs. 12.4%). The usage rates of rectal NSAIDs at doses of 20–25 mg and 50 mg were lower in severe PEP cases (9.2% vs. 12.4% for 20–25 mg and 7.9% vs. 10.8% for 50 mg).

**Table 4:** Comparison of characteristics between severe and non-severe PEP cases

	Non-severe PEP cases (n = 80,371)	Severe PEP cases (n = 4841)	*P* value
Age, mean (SD), years	74.3 (13.3)	75.0 (12.0)	0.002
Sex, male, n (%)	43,151 (53.7)	2847 (58.8)	< 0.001
BMI, mean (SD), kg/m^2a^			
< 18.5, n (%)	9542 (12.5)	675 (14.7)	0.047
≥ 18.5, < 25, n (%)	48,999 (64.2)	2857 (62.4)	0.038
25 < , n (%)	17,814 (23.3)	1049 (22.9)	0.32
CCI			
0, n (%)	38,268 (47.6)	1482 (30.6)	< 0.001
1–2, n (%)	32,152 (40.0)	2127 (43.9)	< 0.001
≥ 3, n (%)	9951 (12.4)	1232 (25.5)	< 0.001
Type of ERCP			
Diagnostic ERCP, n (%)	6880 (8.6)	444 (9.2)	< 0.001
Therapeutic ERCP, n (%)	73,491 (91.4)	4397 (90.8)	< 0.001
Use of rectal NSAIDs, n (%)	19,142 (23.8)	853 (17.7)	< 0.001
12.5 mg, n (%)	502 (0.6)	27 (0.6)	0.52
20–25 mg, n (%)	9998 (12.4)	443 (9.2)	< 0.001
50 mg, n (%)	8642 (10.8)	383 (7.9)	< 0.001
Use of protease inhibitors, n (%)	65,139 (81.1)	3978 (82.2)	0.052
Gabexate mesilate, n (%)^b^	16,581 (20.6)	856 (17.7)	< 0.001
Ulinastatin, n (%)^b^	26,281 (32.7)	1437 (29.7)	< 0.001
Nafamostat mesilate, n (%)^b^	27,119 (33.7)	2089 (43.2)	< 0.001
Use of antibiotics, n (%)	53,526 (66.6)	3220 (66.5)	0.90
PEP-related mortality, n (%)	173 (0.2)	29 (0.6)	< 0.001

*BMI*, body mass index; *CCI*, Charlson Comorbidity Index; *ERCP*, endoscopic retrograde cholangiopancreatography; *NSAIDs*, non-steroidal anti-inflammatory drugs; *PEP*, post-ERCP pancreatitis; *SD*, standard deviation

^a^Data from 76,355 non-severe PEP and 4581 severe PEP cases

^b^Duplicate cases are included

We further analyzed the factors associated with severe PEP. In the multivariate analysis, male sex (OR = 1.17), BMI < 18.5 kg/m^2^ (OR = 1.16), and CCI score of 1–2 (OR = 1.70) and ≥ 3 (OR = 3.11) were associated with increased risk, whereas the use of rectal NSAIDs at doses of 20–25 mg (OR = 0.70) and 50 mg (OR = 0.70) were associated with decreased risk of severe PEP (Online Resource 8). To adjust for patient-related factors, we performed PS matching (Online Resource 9). After PS matching, rectal NSAIDs at doses of 20–25 mg (OR = 0.75) and 50 mg (OR = 0.66) remained significantly associated with a decreased risk of severe PEP (Table 5).

**Table 5:** Univariate analysis and multivariate analysis of risk factors for severe PEP after propensity score matching

	Univariate analysis		Multivariate analysis	
	OR (95% CI)	*P* value	OR (95% CI)	*P* value
Type of ERCP				
Diagnostic ERCP	1		1	
Therapeutic ERCP	0.90 (0.78–1.04)	0.17	0.90 (0.78–1.04)	0.15
NSAIDs				
0 mg	1		1	0.97
12.5 mg	1.01 (0.58–1.74)	0.98	1.01 (0.58–1.74)	0.97
20–25 mg	0.75 (0.65–0.86)	< 0.001	0.75 (0.65–0.86)	< 0.001
50 mg	0.66 (0.57–0.76)	< 0.001	0.66 (0.57–0.76)	< 0.001
Protease inhibitors				
No	1			
Yes	1.04 (0.93–1.15)	0.50		
Antibiotics				
No	1			
Yes	0.99 (0.91–1.08)	0.89		

## Discussion

In this study, we analyzed approximately one million ERCP cases using the DPC database to elucidate the current status and trends in ERCP and PEP, as well as the factors associated with the incidence and severity of PEP. Our findings revealed an increasing trend in the number of ERCP procedures per facility per month and a rising proportion of therapeutic ERCP. In contrast, the incidence of PEP and severe PEP showed a decreasing trend. Regarding PEP prevention, the use of rectal NSAIDs increased, while the use of PIs declined. Notably, rectal NSAIDs at doses of 20–25 mg and 50 mg appeared to prevent the progression of PEP to severe disease. To our knowledge, this is the first study to describe recent trends in ERCP and PEP since the late 2010s, as well as those in rectal NSAIDs and PIs. Our findings provide valuable insights into the evolving landscape of ERCP and PEP management in Japan.

This study revealed an increasing trend in ERCP procedures performed from FY 2016 to FY 2022. Notably, therapeutic ERCP accounted for 90% of all ERCP procedures. While the number of therapeutic ERCP increased, diagnostic ERCP showed a declining trend. These findings align with previous population-based studies in other countries, including the United States, Canada, South Korea, and China [5, 13, 26, 27]. One possible explanation for this trend is the growing prevalence of conditions requiring therapeutic ERCP, such as choledocholithiasis and pancreatobiliary cancers, driven by Japan’s rapidly aging population. These diseases commonly develop in individuals in their 70 s to 80 s [28, 29], and we also observed an upward trend in the age of patients undergoing ERCP. Another contributing factor may be the expansion of ERCP indications due to advances in techniques and devices [13]. The decrease in diagnostic ERCP is likely attributable to improved diagnostic accuracy and the widespread adoption of non-invasive modalities such as magnetic resonance cholangiopancreatography and endoscopic ultrasonography [5, 13, 30]. While a reduction in diagnostic ERCPs could lead to an overall decline in the total number of ERCP procedures, the factors driving an increase in ERCP procedures appear to have had a more pronounced impact in Japan in recent years.

In this study, the incidence of PEP and severe PEP was 7.9% and 0.5%, respectively. These rates are consistent with those reported by Fujita et al. [31], who found similar incidences of 6.9% and 0.5% in a multicenter prospective study on ERCP-related adverse events in Japan. Recent population-based studies have also reported that the incidence of PEP ranges from 4.3 to 16.5% [6, 13, 27]. Many studies have shown an increasing trend in PEP incidence, likely due to the growing number of therapeutic ERCPs and the increasing complexity of cases requiring highly skilled interventions [6, 13]. However, in contrast to these findings, our study demonstrated a decreasing trend in PEP incidence. Several factors may explain this decline. First, preventive strategies for PEP have evolved significantly. Increasing evidence supports the effectiveness of rectal NSAIDs, aggressive hydration, and prophylactic pancreatic stents in preventing PEP [2, 3, 14]. The combined use of these measures has been shown to further reduce PEP risk [32, 33]. Second, the publication of the first clinical practice guidelines for PEP in 2015 [14], along with the revised guidelines for the management of AP in the same year [34], may have increased awareness of PEP risk factors and preventive strategies among Japanese endoscopists. Third, our study focused on the period from 2016 to 2023, whereas previous studies included data only up to 2017 at the latest. This more recent timeframe may better reflect the impact of updated clinical practices. Finally, ERCP in Japan is typically performed in an inpatient setting, which may facilitate the implementation of preventive measures. In contrast, ERCP is often conducted on an outpatient basis in other countries [35], potentially limiting the use of certain preventive strategies.

Our study found that the PEP-related mortality rate was 0.2% among PEP cases, which aligns with findings from a recent systematic review and meta-analysis [4]. Notably, based on our severity criteria, only 14.4% of fatal cases were classified as severe PEP. One possible explanation is that our criteria are stricter than the Japanese severity criteria. In the most recent nationwide epidemiological survey for acute pancreatitis (AP), the proportion of severe cases was similar between all AP cases (23.6%) and PEP cases (23.8%) [36]. However, in our previous study using the DPC database, 27.7% of all AP cases were classified as severe—significantly higher than the proportion of severe PEP cases (5.7%) in this study. This discrepancy suggests that cases classified as severe based on the Japanese criteria may have been categorized as non-severe under our criteria. Another important factor is the advanced age of PEP patients. The mean age of PEP cases in our study was 74.3 years, considerably older than the reported mean age of 61.4 years for all AP cases [19]. In elderly patients, even non-severe PEP may be life-threatening, particularly in those with comorbidities, including malignancies. In fact, malignancies were recorded as the primary disease in 31 (15.3%) of the fatal cases, and among them, 28 (90.3%) were classified as non-severe PEP. It is possible that aggressive treatment, typically indicated for severe cases, was not pursued in these patients due to the presence of malignancies. Unfortunately, for patients whose mortality was attributed to a condition other than the one requiring the highest utilization of medical resources, the exact cause of death remains unclear. We could not rule out that the exact cause of death was unrelated to PEP in these cases.

Several systematic reviews and meta-analyses have demonstrated the effectiveness of rectal NSAIDs in preventing PEP [4, 37, 38]. Accordingly, both the American Society for Gastrointestinal Endoscopy and the European Society of Gastrointestinal Endoscopy recommend their administration in all patients undergoing ERCP [2, 3]. However, in this study, it was unclear whether rectal NSAIDs were used prophylactically for PEP prevention or for pain relief after ERCP, as the DPC database does not provide information on the timing or purpose of their administration. Nevertheless, given the accumulation of recent evidence, the concurrent increase in rectal NSAIDs use, and the decline in PEP incidence, it is reasonable to infer that the increased use of rectal NSAIDs was primarily intended for PEP prevention. Interestingly, rectal NSAIDs use was identified as a factor associated with an increased incidence of PEP. This is likely due to selection bias, as rectal NSAIDs were more frequently administered to patients with high-risk backgrounds and procedural factors associated with PEP. Regarding the severity of PEP, a meta-analysis of eight studies reported that rectal NSAIDs significantly reduced the incidence of moderate-to-severe PEP (OR, 0.53; 95% confidence interval, 0.31–0.89) [39]. However, the preventive effect of low-dose rectal NSAIDs in severe PEP remains unclear. While a 100 mg dose is recommended in Western countries, lower doses (25–50 mg) are predominantly administered in Japan due to smaller body sizes and available dosage forms [40]. Our study revealed a decreasing trend in the incidence of severe PEP, along with an increasing trend in the use of low-dose rectal NSAIDs at doses of 20–25 mg and 50 mg. Furthermore, low-dose rectal NSAIDs use was identified as a factor associated with a lower incidence of severe PEP. To our knowledge, this is the first study to suggest the potential effectiveness of low-dose rectal NSAIDs (20–25 mg and 50 mg) in preventing the progression of PEP to severe disease.

In Japan, PIs have traditionally been used to prevent PEP. Using the public health insurance claims database of the Japan Medical Data Center, Seta et al. [24] reported that the use of PIs in patients undergoing ERCP increased from 72.3% in 2005–2007 to 83.6% in 2010–2015. In contrast, our study demonstrated a decreasing trend in PIs usage, from 70.5% in 2016–2017 to 53.5% in FY 2022. Although our study and Seta et al. [24] utilized different databases, PIs usage may have begun to decline around 2015. This shift may have been influenced by the publication of the Japanese clinical guidelines for PEP in 2015 [14] and the revised guidelines for the management of AP in the same year [34]. The revised AP guidelines explicitly state that drugs other than NSAIDs should not be routinely used for PEP prevention, as their efficacy has been refuted or remains uncertain. They also indicate that the effectiveness of intravenous PIs for AP has not been established. Following these revisions, a similar decline in PIs usage has been observed in AP management overall [19]. The implementation of clinical indicators, known as "pancreatitis bundles," has been associated with reduced mortality in patients with severe AP [41]. These findings highlight the significant impact of clinical guidelines on both decision-making and clinical outcomes. Most recently, the clinical practice guidelines for PEP were updated in 2023 [42], strongly recommending against the use of PIs for PEP prevention. It will be important to monitor whether the prophylactic use of PIs continues to decline in response to these updated recommendations.

This study has several limitations, primarily due to the nature of the DPC database. First, the diagnosis of PEP was based on recorded text data. Additionally, when mortality was attributed to a condition other than the one requiring the highest utilization of medical resources, the exact cause of death could not be determined. Second, the DPC database lacks detailed procedural information related to ERCP, including factors known to influence the risk of PEP, such as precut sphincterotomy, the number of cannulation attempts, and cannulation time. Moreover, it was not possible to determine whether the papilla of Vater was naïve or if the patient had previously undergone ERCP. The DPC database does not allow for patient tracking beyond the current hospitalization, limiting long-term follow-up. Third, medication use was at the discretion of the endoscopist, and information regarding the timing and purpose of rectal NSAIDs and PIs administration was unavailable. The higher usage rate of rectal NSAIDs in PEP cases and their identification as a risk factor for PEP may reflect selection bias, as NSAIDs were likely administered preferentially to high-risk patients. Finally, the database does not capture information on other preventive measures for PEP, such as prophylactic pancreatic stents and aggressive hydration. Despite these limitations, our study provides valuable insights into the current status and temporal trends of ERCP, PEP, and specific preventive measures in Japan.

In conclusion, while the number of ERCP procedures and the proportion of therapeutic ERCPs have been increasing in Japan, the incidences of PEP and severe PEP have shown a declining trend. The usage of rectal NSAIDs has increased, and low-dose rectal NSAIDs (20–25 mg and 50 mg) may help reduce PEP severity. These trends may have been influenced by the publication and implementation of clinical practice guidelines for PEP and AP.

## Supplementary Information

Below is the link to the electronic supplementary material.

Supplementary file1 (DOCX 62 KB)

## Abbreviations

AP: Acute pancreatitis
BMI: Body mass index
CCI: Charlson comorbidity index
DPC: Diagnosis procedure combination
ERCP: Endoscopic retrograde cholangiopancreatography
FY: Fiscal year
ICD-10: International classification of diseases: 10th revision
NSAIDs: Non-steroidal anti-inflammatory drugs
OR: Odds ratio
PEP: Post-ERCP pancreatitis
PIs: Protease inhibitors
PS: Propensity score
SD: Standard deviation
