# Supplementary material for: Current status and trends in ERCP and post-ERCP pancreatitis in Japan: a nationwide observational study
Source: J Gastroenterol. 2025 May 2;60(8):1036–46. doi: 10.1007/s00535-025-02254-8 (PMC12289747; doi:10.1007/s00535-025-02254-8)
Supplement: Supplementary file 1 — Supplementary file1 (DOCX 62 KB) [file 535_2025_2254_MOESM1_ESM.docx]

***Journal of Gastroenterology***

**Current status and trends in ERCP and post-ERCP pancreatitis in Japan: a nationwide observational study**

Tomoo Manaka, Tetsuya Takikawa, Kunio Tarasawa, Kazuhiro Kikuta, Ryotaro Matsumoto, Yu Tanaka, Takanori Sano, Shin Hamada, Shin Miura, Kiyoshi Kume, Kenji Fujimori, Kiyohide Fushimi, Atsushi Masamune

**Corresponding author:**

Tetsuya Takikawa, MD, PhD,

Division of Gastroenterology,

Tohoku University Graduate School of Medicine,

1-1 Seiryo-machi, Aoba-ku, Sendai 980-8574, Japan

Tel.: +81-22-717-7171; Fax: +81-22-717-7177

E-mail: tetsuya.takikawa.c2@tohoku.ac.jp

| **Online Resource 1. Procedure codes and ERCP procedure** | | |
| --- | --- | --- |
| **Type of ERCP type** | **Procedure code** | **Procedures** |
| Diagnostic | 160093970, 170015670 | Cholangiography and pancreatography |
|  | 160161170, 170020370 | Cholangioscopy and pancreatoscopy |
| Therapeutic | 150362310 | Endoscopic nasobiliary drainage |
|  | 150174910  150362510  150437770 | Endoscopic biliary stone removal with biliary lithotripsy  Endoscopic biliary stone removal (others)  Endoscopic biliary stone removal using balloon endoscopy |
|  | 150175310  150437670 | Endoscopic biliary dilation  Endoscopic biliary dilation using balloon endoscopy  Balloon-assisted endoscopic biliary dilatation |
|  |  |  |
|  | 150175410  150296710  150417510  150437970 | Endoscopic sphincterotomy  Endoscopic sphincterotomy with biliary lithotripsy  Endoscopic sphincterotomy with biliary lithotripsy under cholangioscopy  Endoscopic sphincterotomy using balloon endoscopy |
|  | 150254410  150437870 | Endoscopic biliary stenting  Endoscopic biliary stenting using balloon endoscopy |
|  | 150363610 | Endoscopic pancreatic stenting |
| *ERCP*, endoscopic retrograde cholangiopancreatography | | |

| **Online Resource 2. Primary diagnosis and causes of mortality** | | | |
| --- | --- | --- | --- |
| Mortality due to the condition that required the highest utilization of medical resources | | | |
| Cause of mortality | Non-severe PEP  (n = 89) | Severe PEP  (n = 15) | Total PEP  (n = 104) |
| PEP, n (%) | 78 (87.6) | 7 (46.7) | 85 (81.7) |
| Acute pancreatitis, n (%) | 6 (6.7) | 8 (53.3) | 14 (13.5) |
| DIC, n (%) | 2 (2.2) | 0 (0) | 2 (1.9) |
| Sepsis, n (%) | 2 (2.2) | 0 (0) | 2 (1.9) |
| Pancreatic pseudocyst, n (%) | 1 (1.1) | 0 (0) | 1 (1.0) |
|  |  |  |  |
| Mortality due to a condition other than that required the highest utilization of medical resources | | | |
| Primary diagnosis | Non-severe PEP  (n = 84) | Severe PEP  (n = 14) | Total PEP  (n = 98) |
| PEP, n (%) | 47 (56.0) | 7 (50.0) | 54 (55.1) |
| Acute pancreatitis, n (%) | 3 (3.6) | 4 (28.6) | 7 (7.1) |
| Bile duct cancer, n (%) | 14 (16.7) | 1 (7.1) | 15 (15.3) |
| Cholangitis, n (%) | 8 (9.5) | 1 (7.1) | 9 (9.2) |
| Cholecystitis, n (%) | 4 (4.8) | 0 (0) | 4 (4.1) |
| Pancreatic cancer, n (%) | 3 (3.6) | 0 (0) | 3 (3.1) |
| Sepsis, n (%) | 1 (1.2) | 0 (0) | 1 (1.0) |
| Choledocholithiasis, n (%) | 1 (1.2) | 0 (0) | 1 (1.0) |
| Obstructive jaundice, n (%) | 1 (1.2) | 0 (0) | 1 (1.0) |
| Gastric cancer, n (%) | 1 (1.2) | 0 (0) | 1 (0.5) |
| Lung cancer, n (%) | 1 (1.2) | 0 (0) | 1 (0.5) |
| Takotsubo cardiomyopathy, n (%) | 0 (0) | 1 (7.1) | 1 (0.5) |
| *PEP*, post-ERCP pancreatitis; *DIC*, disseminated intravascular coagulation | | | |

| **Online Resource 3. Characteristics of PEP cases (n = 85,212)** | |
| --- | --- |
|  | **PEP cases** |
| Age, mean (SD), years | 74.3 (13.2) |
| Sex, Male, n (%) | 45,998 (54.0) |
| BMI (kg/m^2^) ^a^ |  |
| < 18.5, n (%) | 10,217 (12.6) |
| ≥ 18.5, < 25, n (%) | 51,856 (64.1) |
| ≥ 25, n (%) | 18,863 (23.3) |
| CCI |  |
| 0, n (%) | 39,750 (46.7) |
| 1–2, n (%) | 34,279 (40.2) |
| ≥ 3, n (%) | 11,183 (13.1) |
| ERCP type |  |
| Diagnostic ERCP, n (%) | 7,324 (8.6) |
| Therapeutic ERCP, n (%) | 77,888 (91.4) |
| Use of rectal NSAIDs, n (%) | 19,995 (23.5) |
| 12.5mg, n (%) | 529 (0.6) |
| 20-25mg, n (%) | 10,441 (12.3) |
| 50mg, n (%) | 9,025 (10.6) |
| Use of protease inhibitors, n (%) | 69,117 (81.1) |
| Gabexate mesilate, n (%) ^b^ | 17,437 (20.5) |
| Ulinastatin, n (%) ^b^ | 27,718 (32.5) |
| Nafamostat mesilate, n (%) ^b^ | 29,208 (34.3) |
| Use of antibiotics, n (%) | 56,746 (66.6) |
| Severe PEP, n (%) | 4,841 (5.7) |
| PEP-related mortality, n (%) | 202 (0.2) |
| ^a^ Data from 80,936 cases. ^b^ Duplicate cases are included.  *BMI*, body mass index; *CCI*, Charlson Comorbidity Index; *ERCP*, endoscopic retrograde cholangiopancreatography; *NSAIDs*, non-steroidal anti-inflammatory drugs; *PEP*, post-ERCP pancreatitis; *SD*, standard deviation | |

| **Online Resource 4. Trends in ERCP among PEP cases from 2016 to 2022** | | | | | |
| --- | --- | --- | --- | --- | --- |
| **Fiscal year** | **2016–2017** | **2018–2019** | **2020–2021** | **2022** | ***P* value**  **for trend** |
| The number of PEP, n | 27,493 | 24,569 | 23,340 | 9,810 | - |
| Age, mean (SD), years |  |  |  |  |  |
| Total | 73.7 (13.2) | 74.2 (13.2) | 74.8 (13.3) | 75.2 (13.1) | < 0.001 |
| Male | 72.5 (12.3) | 73.0 (12.5) | 73.6 (12.6) | 73.9 (12.4) | < 0.001 |
| Female | 75.1 (14.0) | 75.6 (13.8) | 76.3 (13.9) | 76.7 (13.7) | < 0.001 |
| Sex, Male, n (%) | 14,924 (54.3) | 13,154 (53.5) | 12,637 (54.1) | 5,283 (53.9) | 0.63 |
| BMI (kg/m^2^) ^a^ |  |  |  |  |  |
| < 18.5, n (%) | 3,405 (13.0) | 2,913 (12.4) | 2,678 (12.1) | 1,221 (13.2) | 0.004 |
| ≥ 18.5, < 25, n (%) | 16,847 (64.5) | 14,986 (64.0) | 14,137 (63.8) | 5,886 (63.6) | 0.06 |
| ≥ 25, n (%) | 5,862 (22.5) | 5,515 (23.6) | 5,333 (24.1) | 2,153 (23.2) | < 0.001 |
| CCI |  |  |  |  |  |
| 0, n (%) | 12,593 (45.8) | 11,439 (46.6) | 11,026 (47.2) | 4,692 (47.8) | < 0.001 |
| 1–2, n (%) | 11,102 (40.4) | 9,864 (40.1) | 9,315 (39.9) | 3,998 (40.8) | 0.93 |
| ≥ 3, n (%) | 3,798 (13.8) | 3,266 (13.3) | 2,999 (12.9) | 1,120 (11.4) | < 0.001 |
| ERCP type |  |  |  |  |  |
| Diagnostic ERCP, n (%) | 2,843 (10.3) | 2,143 (8.7) | 1,722 (7.4) | 616 (6.3) | < 0.001 |
| Therapeutic ERCP, n (%) | 24,650 (89.7) | 22,426 (91.3) | 21,618 (92.6) | 9,194 (93.7) | < 0.001 |
| Use of rectal NSAIDs, n (%) | 4,858 (17.7) | 5,774 (23.5) | 6,449 (27.6) | 2,914 (29.7) | < 0.001 |
| 12.5mg, n (%) | 180 (0.65) | 159 (0.64) | 135 (0.58) | 55 (0.56) | 0.07 |
| 20–25mg, n (%) | 2,504 (9.1) | 2,918 (11.9) | 3,341 (14.3) | 1,678 (17.1) | < 0.001 |
| 50mg, n (%) | 2,174 (7.9) | 2,697 (11.0) | 2,973 (12.7) | 1,181 (12.0) | < 0.001 |
| Use of protease inhibitors, n (%) | 23,602 (85.8) | 19,992 (81.4) | 18,309 (78.4) | 7,214 (73.5) | < 0.001 |
| Gabexate mesilate, n (%) ^b^ | 7,143 (26.0) | 4,593 (18.7) | 4,013 (17.2) | 1,688 (17.2) | < 0.001 |
| Ulinastatin, n (%) ^b^ | 9,351 (34.0) | 7,859 (32.0) | 7,511 (32.2) | 2,997 (30.6) | < 0.001 |
| Nafamostat mesilate, n (%) ^b^ | 9,158 (33.3) | 9,073 (36.9) | 8,019 (34.4) | 2,958 (30.2) | < 0.001 |
| Use of antibiotics, n (%) | 16,938 (61.6) | 16,664 (67.8) | 16,121 (69.1) | 7,023 (71.6) | < 0.001 |
| Severe PEP, n (%) | 1,508 (5.5) | 1,619 (6.6) | 1,212 (5.2) | 502 (5.1) | 0.013 |
| PEP-related mortality, n (%) | 44 (0.16) | 73 (0.30) | 60 (0.26) | 25 (0.25) | 0.041 |
| ^a^ Data from 80,936 cases. ^b^ Duplicate cases are included.  *BMI*, body mass index; *CCI*, Charlson Comorbidity Index; *ERCP*, endoscopic retrograde cholangiopancreatography; *NSAIDs*, non-steroidal anti-inflammatory drugs; *PEP*, post-ERCP pancreatitis; *SD*, standard deviation | | | | | |

| **Online Resource 5. Univariate analysis and multivariate analysis of risk factors for PEP** | | | | | |
| --- | --- | --- | --- | --- | --- |
|  | **Univariate analysis** | |  | **Multivariate analysis** | |
|  | **OR (95% CI)** | ***P* value** |  | **OR (95% CI)** | ***P* value** |
| **Age (years)** |  |  |  |  |  |
| < 65 | 1 |  |  | 1 |  |
| ≥ 65 | 1.05 (1.03–1.07) | < 0.001 |  | 1.07 (1.05–1.09) | < 0.001 |
| **Sex** |  |  |  |  |  |
| Female | 1 |  |  | 1 |  |
| Male | 0.83 (0.82–0.84) | < 0.001 |  | 0.83 (0.82–0.85) | < 0.001 |
| **BMI (kg/m^2^)** |  |  |  |  |  |
| < 18.5 | 0.89 (0.87-0.91) | < 0.001 |  | 0.88 (0.86-0.90) | < 0.001 |
| ≥ 18.5, <25 | 1 |  |  | 1 |  |
| ≥ 25 | 1.08 (1.07–1.10) | < 0.001 |  | 1.07 (1.05–1.09) | < 0.001 |
| **CCI** |  |  |  |  |  |
| 0 | 1 |  |  | 1 |  |
| 1–2 | 0.95 (0.94–0.97) | < 0.001 |  | 0.96 (0.94–0.97) | < 0.001 |
| ≥ 3 | 0.89 (0.87–0.90) | < 0.001 |  | 0.92 (0.90–0.95) | < 0.001 |
| **ERCP type** |  |  |  |  |  |
| Diagnostic ERCP | 1 |  |  | 1 |  |
| Therapeutic ERCP | 1.20 (1.17–1.23) | < 0.001 |  | 1.17 (1.14–1.20) | < 0.001 |
| **Rectal NSAIDs** |  |  |  |  |  |
| 0mg | 1 |  |  | 1 |  |
| 12.5mg | 1.62 (1.47–1.76) | < 0.001 |  | 1.59 (1.44–1.74) | < 0.001 |
| 20–25mg | 1.18 (1.15–1.21) | < 0.001 |  | 1.26 (1.23–1.29) | < 0.001 |
| 50mg | 1.02 (0.99–1.05) | 0.060 |  | 1.17 (1.14–1.20) | < 0.001 |
| **Protease inhibitors** |  |  |  |  |  |
| No | 1 |  |  | 1 |  |
| Yes | 2.65 (2.60–2.70) | < 0.001 |  | 2.70 (2.65–2.75) | < 0.001 |
| **Antibiotics** |  |  |  |  |  |
| No | 1 |  |  | 1 |  |
| Yes | 0.97 (0.95–0.98) | < 0.001 |  | 0.89 (0.88–0.91) | < 0.001 |
| *BMI*, body mass index; *CCI*, Charlson Comorbidity Index; *CI*, confidence interval; *ERCP*, endoscopic retrograde cholangiopancreatography; *NSAIDs*, non-steroidal anti-inflammatory drugs; *OR*, odds ratio; *PEP*, post-ERCP pancreatitis | | | | | |

| **Online Resource 6. Comparison of characteristics between PEP and non-PEP cases before and after propensity score matching** | | | | | | | | | |
| --- | --- | --- | --- | --- | --- | --- | --- | --- | --- |
|  | **Before propensity score matching** | | | |  | **After propensity score matching** | | | |
|  | **Non-PEP**  **(n=988,301)** | **PEP**  **(n=85,212)** | ***P* value** | **ASD** |  | **Non-PEP**  **(n=80,936)** | **PEP**  **(n=80,936)** | ***P* value** | **ASD** |
| **Age, years** |  |  | < 0.001 | 0.02 |  |  |  | 1.00 | < 0.001 |
| < 65, n (%) | 192,618 (19.5) | 15,949 (18.7) |  |  |  | 15,265 (18.9) | 15,265 (18.9) |  |  |
| ≥ 65, n (%) | 795,683 (80.5) | 69,263 (81.3) |  |  |  | 65,671 (81.1) | 65,671 (81.1) |  |  |
| **Sex** |  |  | < 0.001 | 0.09 |  |  |  | 1.00 | < 0.001 |
| Female, n (%) | 410,038 (41.5) | 39,214 (46.0) |  |  |  | 36,972 (45.7) | 36,972 (45.7) |  |  |
| Male, n (%) | 578,263 (58.5) | 45,998 (54.0) |  |  |  | 43,964 (54.3) | 43,964 (54.3) |  |  |
| **BMI, kg/m^2 a^** |  |  | < 0.001 | 0.06 |  |  |  | 1.00 | < 0.001 |
| < 18.5, n (%) | 13,4043 (14.3) | 10,217 (12.6) |  |  |  | 10,217 (12.6) | 10,217 (12.6) |  |  |
| ≥ 18.5, < 25, n (%) | 602,920 (64.2) | 51,856 (64.1) |  |  |  | 51,856 (64.1) | 51,856 (64.1) |  |  |
| ≥ 25, n (%) | 202,156 (21.5) | 18,863 (23.3) |  |  |  | 18,863 (23.3) | 18,863 (23.3) |  |  |
| **CCI** |  |  | < 0.001 | 0.04 |  |  |  | 1.00 | < 0.001 |
| 0, n (%) | 444,272 (44.9) | 39,750 (46.7) |  |  |  | 37,840 (46.8) | 37,840 (46.8) |  |  |
| 1–2, n (%) | 402,852 (40.8) | 34,279 (40.2) |  |  |  | 32,283 (40.0) | 32,283 (40.0) |  |  |
| ≥ 3, n (%) | 141,177 (14.3) | 11,183 (13.1) |  |  |  | 10,713 (13.2) | 10,713 (13.2) |  |  |
| ^a^ Data from 939,119 non-PEP and 80,936 PEP cases before propensity score matching.  *ASD,* absolute standardized difference; *BMI*, body mass index; *CCI*, Charlson Comorbidity Index; *PEP*, post-ERCP pancreatitis | | | | | | | | | |

| **Online Resource 7. Univariate and multivariate analysis of risk factors for PEP after propensity score matching** | | | | | |
| --- | --- | --- | --- | --- | --- |
|  | **Univariate analysis** | |  | **Multivariate analysis** | |
|  | **OR (95% CI)** | ***P* value** |  | **OR (95% CI)** | ***P* value** |
| **ERCP type** |  |  |  |  |  |
| Diagnostic ERCP | 1 |  |  | 1 |  |
| Therapeutic ERCP | 3.63 (3.53–3.74) | < 0.001 |  | 3.63 (3.52–3.74) | < 0.001 |
| **Rectal NSAIDs** |  |  |  |  |  |
| 0mg | 1 |  |  | 1 |  |
| 12.5mg | 1.89 (1.63–2.19) | < 0.001 |  | 1.64 (1.41–1.91) | < 0.001 |
| 20–25mg | 1.08 (1.05–1.11) | < 0.001 |  | 1.18 (1.14–1.21) | < 0.001 |
| 50mg | 1.12 (1.08–1.16) | < 0.001 |  | 1.27 (1.22–1.31) | < 0.001 |
| **Protease inhibitors** |  |  |  |  |  |
| No | 1 |  |  | 1 |  |
| Yes | 2.52 (2.47–2.58) | < 0.001 |  | 2.30 (2.25–2.35) | < 0.001 |
| **Antibiotics** |  |  |  |  |  |
| No | 1 |  |  | 1 |  |
| Yes | 1.86 (1.83–1.90) | < 0.001 |  | 1.74 (1.71–1.78) | < 0.001 |
| *CI*, confidence interval; *ERCP*, endoscopic retrograde cholangiopancreatography; *NSAIDs*, non-steroidal anti-inflammatory drugs; *OR*, odds ratio; *PEP*, post-ERCP pancreatitis | | | | | |

| **Online Resource 8. Univariate analysis and multivariate analysis of risk factors for severe PEP** | | | | | |
| --- | --- | --- | --- | --- | --- |
|  | **Univariate analysis** | |  | **Multivariate analysis** | |
|  | **OR (95% CI)** | ***P* value** |  | **OR (95% CI)** | ***P* value** |
| **Age (years)** |  |  |  |  |  |
| < 65 | 1 |  |  | 1 |  |
| ≥ 65 | 1.20 (1.11–1.30) | < 0.001 |  | 1.05 (0.97–1.14) | 0.22 |
| **Sex** |  |  |  |  |  |
| Female | 1 |  |  | 1 |  |
| Male | 1.23 (1.16–1.31) | < 0.001 |  | 1.17 (1.10–1.25) | < 0.001 |
| **BMI (kg/m^2^)** |  |  |  |  |  |
| < 18.5 | 1.21 (1.11-1.32) | < 0.001 |  | 1.16 (1.06-1.27) | 0.001 |
| ≥ 18.5, <25 | 1 |  |  | 1 |  |
| ≥ 25 | 1.01 (0.94–1.09) | 0.79 |  | 1.06 (0.98–1.14) | 0.15 |
| **CCI** |  |  |  |  |  |
| 0 | 1 |  |  | 1 |  |
| 1–2 | 1.71 (1.60–1.83) | < 0.001 |  | 1.70 (1.59–1.83) | < 0.001 |
| ≥ 3 | 3.20 (2.95–3.46) | < 0.001 |  | 3.11 (2.86–3.37) | < 0.001 |
| **ERCP type** |  |  |  |  |  |
| Diagnostic ERCP | 1 |  |  | 1 |  |
| Therapeutic ERCP | 0.93 (0.84–1.03) | 0.14 |  | 0.91 (0.82–1.01) | 0.076 |
| **Rectal NSAIDs** |  |  |  |  |  |
| 0mg | 1 |  |  | 1 |  |
| 12.5mg | 0.83 (0.56-1.22) | 0.83 |  | 0.87 (0.59-1.29) | 0.49 |
| 20–25mg | 0.68 (0.62–0.75) | < 0.001 |  | 0.70 (0.63–0.77) | < 0.001 |
| 50mg | 0.68 (0.61–0.76) | < 0.001 |  | 0.70 (0.62–0.78) | < 0.001 |
| **Protease inhibitors** |  |  |  |  |  |
| No | 1 |  |  | 1 |  |
| Yes | 1.08 (1.00–1.16) | 0.052 |  | 1.01 (0.94–1.10) | 0.75 |
| **Antibiotics** |  |  |  |  |  |
| No | 1 |  |  |  |  |
| Yes | 1.00 (0.94–1.06) | 0.90 |  |  |  |
| *BMI*, body mass index; *CCI*, Charlson Comorbidity Index; *CI*, confidence interval; *ERCP*, endoscopic retrograde cholangiopancreatography; *NSAIDs*, non-steroidal anti-inflammatory drugs; *OR*, odds ratio; *PEP*, post-ERCP pancreatitis | | | | | |

| **Online Resource 9. Comparison** **of characteristics between severe and non-severe PEP before and after propensity score matching** | | | | | | | | | |
| --- | --- | --- | --- | --- | --- | --- | --- | --- | --- |
|  | **Before propensity score matching** | | | |  | **After propensity score matching** | | | |
|  | **Non-severe PEP (n=80,371)** | **Severe PEP (n=4,841)** | ***P* value** | **ASD** |  | **Non-severe PEP (n=4,581)** | **Severe PEP (n=4,581)** | ***P* value** | **ASD** |
| **Age, years** |  |  | < 0.001 | 0.07 |  |  |  | 1.00 | < 0.001 |
| < 65, n (%) | 15,163 (18.9) | 786 (16.2) |  |  |  | 744 (16.2) | 744 (16.2) |  |  |
| ≥ 65, n (%) | 65,208 (81.1) | 4,055 (83.8) |  |  |  | 3,837 (83.8) | 3,837 (83.8) |  |  |
| **Sex** |  |  | < 0.001 | 0.10 |  |  |  | 1.00 | < 0.001 |
| Female, n (%) | 37,220 (46.3) | 1,994 (41.2) |  |  |  | 1,875 (40.9) | 1,875 (40.9) |  |  |
| Male, n (%) | 43,151 (53.7) | 2,847 (58.8) |  |  |  | 2,706 (59.1) | 2,706 (59.1) |  |  |
| **BMI, kg/m^2 a^** |  |  | < 0.001 | 0.07 |  |  |  | 1.00 | < 0.001 |
| < 18.5, n (%) | 9,542 (12.5) | 675 (14.7) |  |  |  | 675 (14.7) | 675 (14.7) |  |  |
| ≥ 18.5, < 25, n (%) | 48,999 (64.2) | 2,857 (62.4) |  |  |  | 2,857 (62.4) | 2,857 (62.4) |  |  |
| ≥ 25, n (%) | 17,814 (23.3) | 1,049 (22.9) |  |  |  | 1,049 (22.9) | 1,049 (22.9) |  |  |
| **CCI** |  |  | < 0.001 | 0.43 |  |  |  | 1.00 | < 0.001 |
| 0, n (%) | 38,268 (47.6) | 1,482 (30.6) |  |  |  | 1,391 (30.4) | 1,391 (30.4) |  |  |
| 1–2, n (%) | 32,152 (40.0) | 2,127 (43.9) |  |  |  | 2,014 (44.0) | 2,014 (44.0) |  |  |
| ≥ 3, n (%) | 9,951 (12.4) | 1,232 (25.5) |  |  |  | 1,176 (25.7) | 1,176 (25.7) |  |  |
| ^a^ Data from 76,355 non-severe PEP and 4,581 severe PEP cases before propensity score matching.  *ASD,* absolute standardized difference; *BMI*, body mass index; *CCI*, Charlson Comorbidity Index; *PEP*, post-ERCP pancreatitis | | | | | | | | | |
